# One-Step Synthesis of Nitrogen/Fluorine Co-Doped Carbon Dots for Use in Ferric Ions and Ascorbic Acid Detection

**DOI:** 10.3390/nano12142377

**Published:** 2022-07-12

**Authors:** Yan Zhao, Xiaoxuan Zhu, Lu Liu, Zhiqing Duan, Yanping Liu, Weiyuan Zhang, Jingjing Cui, Yafang Rong, Chen Dong

**Affiliations:** 1 School of Chemical Engineering, Hebei Normal University of Science and Technology, Qinhuangdao 066004, China; zhuxiaoxuan1999@163.com (X.Z.); 13633336800@163.com (L.L.); qizixuanyuanke@163.com (Z.D.); lyp3893@hevttc.edu.cn (Y.L.); 13649502263@163.com (W.Z.); cuijingjing23@outlook.com (J.C.); 2 Hebei Key Laboratory of Active Components and Functions in Natural Product, Qinhuangdao 066004, China; 3 Shandong Zhengyuan Geophysical Information Technology Co., Ltd., Jinan 250000, China; 13953173230@139.com; 4 Cixi Institute of Biomedical Engineering, International Cooperation Base of Biomedical Materials Technology and Application, CAS Key Laboratory of Magnetic Materials and Devices, Zhejiang Engineering Research Center for Biomedical Materials, Ningbo Institute of Materials Technology and Engineering, Chinese Academy of Sciences, Ningbo 315201, China

**Keywords:** carbon dots, nitrogen and fluorine-doped, fluorescent sensor, ferric ion, ascorbic acid

## Abstract

Carbon dots (CDs) have caught enormous attention owing to their distinctive properties, such as their high water solubility, tunable optical properties, and easy surface modification, which can be generally used for the detection of heavy metals and organic pollutants. Herein, nitrogen and fluorine co-doped carbon dots (NFCDs) were designed via a rapid, low-cost, and one-step microwave-assisted technique using DL-malic acid and levofloxacin. The NFCDs emitted intense green fluorescence under UV lighting, and the optical emission peak at 490 nm was observed upon a 280 nm excitation, with a high quantum yield of 21.03%. Interestingly, the spectral measurements illustrated excitation-independent and concentration-independent single-color fluorescence owing to the presence of nitrogen and fluorine elements in the surface functional groups. Additionally, the NFCDs were applied for the selective detection of Fe^3+^ and ascorbic acid based on the “turn-off” mode. The detection limits were determined as 1.03 and 4.22 µM, respectively. The quenching mechanisms were explored using the static quenching mechanism and the inner filter effect. Therefore, a NFCDs fluorescent probe with single color emission was successfully developed for the convenient and rapid detection of Fe^3+^ and ascorbic acid in environments.

## 1. Introduction

With the development of chemical industries, water pollution caused by heavy metal ions and organic pollutants has become a major environmental and public health issue. Ferric ion (Fe^3+^) is a transition metal that plays a vital role in biogeochemical processes [1,2]. As the fourth abundant element in the Earth’s crust, iron can be found in both surface and underground waters. Moreover, the various applications of Fe-related industrial processes, such as sewage treatment systems and a Fenton reagent, would further increase the amount of iron in environments and thus cause potential hazards to human health [3,4]. It has been widely recognized that a high concentration of Fe^3+^ is associated with various health implications, including cancers of different organs, cellular homeostasis, or metabolism [4,5,6,7]. Additionally, the permissible amount of ferric ions in drinking waters was set at 300 ppb by the Environmental Protection Agency [1,8]. Therefore, the ferric ion level is one important property to consider when evaluating the quality of water.

On the other hand, ascorbic acid (AA), also named vitamin C, is one of the most important water-soluble vitamins in the human body. The excess or lack of AA would reduce the metabolism and redox and thus lead to various diseases [9,10]. Generally, the fluorescent materials applied for metal ions and AA detection are divided into several groups, including gold and silver nanoclusters [11,12], organic dyes [13,14], and so on [15,16]. However, most of the materials have suffered from sophisticated material preparation procedures, high toxicity, and poor water solubility, especially when dealing with living systems. Therefore, it is of great importance and urgency to design a novel fluorescent sensor with a highly sensitive response, facile and green synthesis approach, and excellent optical properties.

In the past few years, great efforts have been paid to develop fluorescence probes for the detection of heavy metals or organic molecules in environments. Among all the nanomaterials recently designed for sensing applications, carbon dots (CDs), a typical member of the carbon materials family, have attracted enormous attention in the field of fluorescence sensors, owing to their excellent optical properties, good biocompatibility, high water solubility, and environmental friendliness [5,17,18,19,20,21]. Recently, various applications of CDs in environmental monitoring and chemical sensing have been explored [4,5,22,23,24,25]. It is widely known that the carbon cores and surface structure of CDs are highly related to their optical properties and detection capability [5,26,27,28]. Thus, as a popular modification approach, the heteroatom doping on the surface of the functional groups of CDs can be further used to tune the emissive properties and thus improve the quantum yield (QY), such as nitrogen (N), fluorine (F), sulfur (S), phosphorus (P), silicon (Si) and boron (B) [29,30,31,32,33,34,35]. For example, N-doped CDs were successfully constructed using a solvothermal approach for the early detection of Fe^3+^ during iron corrosion in a NaCl solution. The limit of detection (LOD) was estimated to be 0.9 µM [5]. Zhang and colleagues prepared boron and nitrogen co-doped CDs (QY as 15.4%) and successfully used them for the detection of Fe^3+^ with a LOD of 0.08 µM [17], whereas Liu et al. synthesized N/S-doped CDs with an improved QY of 26% [36]. Furthermore, Li and colleagues applied a cobalt-doped CD for the detection of Fe^3+^ and AA, respectively. The fluorescence of CDs was firstly turned off by the Fe^3+^ ions, which were further rebuilt (off–on) after the addition of AA, following an “on-off-on” mode. The Co-CDs showed an enhanced QY of 30.4% with a blue emission upon a 350 nm excitation [26]. Cui et al. found that the N-doped CDs can be directly quenched by AA—as a “turn-off” probe—with a LOD of 2.6 µM. From the PL lifetime and UV spectral analyses, the NCDs/Fe^3+^ and NCDs/AA showed the formation of a new non-fluorescence complex, referring to the static quenching mechanism (SQM). Meanwhile, NCDs’ excitation spectra were found to overlap with the UV spectra of Fe^3+^ and AA, indicating the absorption of excitation light, which is named the inner filter effects (IFE). The quenching mechanism was further explored as the IFE and SQM [37].

Furthermore, it has been demonstrated that the dopant atoms on the surface functional groups affect the electronic transition significantly, indicating a promising strategy for preparing nanomaterials with specific or tunable fluorescence emissions [38]. Among all the commonly used elements for doping, the introduction of F presents the electron-withdrawing effects and strong hydrogen bonding interactions, which would change the surface charge, tune the emission fluorescence and thus improve the detection capacities [39,40,41,42]. In our previous work, N-doped DL-malic acid-derived CDs were synthesized with blue fluorescence and a QY of 9.8% [37].

**Scheme 1:**
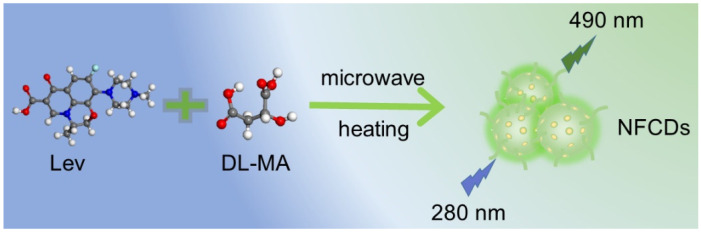
The preparation approach of NFCDs from Lev and DL-MA.

Therefore, in order to tune the optical and physical properties of the CDs, novel DL-malic acid (DL-MA)-derived carbon dots were prepared using levofloxacin (Lev) as the F source with an efficient microwave-assisted approach (as seen in Scheme 1). The designed N- and F-doped CDs (NFCDs) were further characterized by high-resolution transmission element microscopy (HR-TEM), X-ray diffraction (XRD), Fourier transform infrared spectroscopy (FT-IR), and X-ray photoelectron spectroscopy (XPS) to confirm the morphology and surface functional groups. In addition, the NFCDs were found to emit a green fluorescence under the UV lamp, with a photoluminescence quantum yield (QY) detected as 21.03%. The optimal emission wavelengths of the NFCDs were observed at 490 nm upon a 280 nm excitation, independent of the excitation wavelengths and concentration. The green fluorescence can be quenched by both Fe^3+^ and AA, respectively. The as-prepared NFCDs were further used for the detection of Fe^3+^ and AA, demonstrating LODs of 1.03 and 4.22 µM, respectively. The LODs are comparable with recent research [35,36,37,38,39]. Therefore, the N and F co-doped CDs with green fluorescence were successfully synthesized with an excitation-independent emission, high QY, and high selectivity and sensitivity for the detection of Fe^3+^ and AA.

## 2. Materials and Methods

### 2.1. Materials

DL-malic acid and levofloxacin were purchased from Aladdin Biochemical Technology Co., Ltd., (Shanghai, China). All the inorganic reagents, including sodium chloride, potassium chloride, aluminum nitrate nonahydrate, manganese chloride tetrahydrate, ferric chloride, magnesium chloride hexahydrate, nickel chloride hexahydrate, cobalt chloride hexahydrate, calcium chloride, stannous chloride, lead (II) chloride, and barium chloride dehydrate, were of analytical grade and obtained from Sinopharm Chemical Reagent Co., Ltd., (Beijing, China). All the organic compounds, such as D-asparagine (D-Asn), L-glutamic acid (LGA), L-valine (L-Val), L-methionine (L-Met), L-asparagine (L-Asn), L-leucine (L-Leu), S-mandelic acid (S-MA), L-glutamine (L-Glu), and L-ascorbic acid (AA) were supplied by Aladdin Industrial Corporation. The ultrapure water used was directly obtained from the lab with a Milli-Q direct water purification system.

### 2.2. Synthesis of NFCDs

Firstly, the desired amounts of DL-malic acid and levofloxacin were dissolved in 10 mL of ultrapure water, which was then heated in a microwave oven (600 W, M1-L213B, Media, China) for 5 min. The resultant solids were dissolved in water with an ultrasound treatment (40 KHz, F-040, Fuyang Technology group Co., Ltd., Shenzhen, China) for around 24 h and then filtered with a filtration membrane (0.22 μm). The obtained solution was further purified with dialysis bags (MDCO 34-1000 Da, Biomed Instrument Inc., USA) for 24 h. Eventually, the purified NFCDs solution was obtained and further freeze-dried at −70 °C for further characterization.

### 2.3. Characterization of NFCDs

The morphology of the NFCDs was analyzed by transmission electron microscopy (TEM, JEM-2100, JEOL Corp., Japan). A FIR8900 Fourier transform infrared (FT-IR) spectrometer and X-ray photoelectron spectroscopy (XPS, Escalab 250Xi, Thermo Scientific, Waltham, MA, USA) were used to investigate the surface groups of the NFCDs. The X-ray diffraction (XRD) pattern of the NFCDs was obtained from the Neotoku Corporation (Japan) with CuKα1 radiation operated at 40 kV and 40 mA. For the optical properties analysis, the UV-vis absorbance spectra and fluorescence light (PL) spectra were collected on a U-4100 spectrophotometer (Hitachi, Tokyo, Japan) and Hitachi F-7000 fluorescence spectrometer (150 W xenon lamp), respectively. The PL decay lifetimes were measured and estimated based on an FLS920 spectrophotometer supplied by Edinburgh Instruments. The absolute quantum yield (QY) was measured in the calibration sphere with a HORIBA FLS920.

### 2.4. Stability of NFCDs Solution

In order to examine the photostability of the NFCDs, the solution was continuously exposed to UV light (365 nm) for 2 h. Upon 280 nm excitation, the PL spectra were recorded every 10 min. Moreover, the concentration-dependent PL property of the NFCDs-1 was explored by collecting the PL spectra of solutions with different concentrations, ranging from 0.005 to 0.5 mg/mL. Additionally, the effects of the acidic or basic environments on the emission spectra (upon 280 nm excitation) were further investigated with different pH values (ranging from 2.2 to 13.1), which were tuned using a NaOH solution (0.1 mol/L), HCl solution (0.1 mol/L), and NaH_2_PO_3_ solution (0.01 mol/L).

### 2.5. Fluorescence Quenching Study of Fe^3+^ and AA

For the detection of Fe^3+^ and AA, based on the “turn-off” mode, the stock solution of the NFCDs was mixed with different amounts of FeCl_3_ solution and AA solution. For the detection of Fe^3+^, solutions with different Fe^3+^ concentrations ranging from 5 to 500 µM were prepared in 0.02 mg/mL NFCDs. On the other hand, the concentration of AA was set from 5 to 900 µM. After mixing for 5 min, the PL spectra of different solutions were recorded upon a 280 nm excitation. All the experiments were conducted in triplicate.

## 3. Results and Discussion

### 3.1. Morphology and Structure Characterization of NFCDs

**Figure 1:**
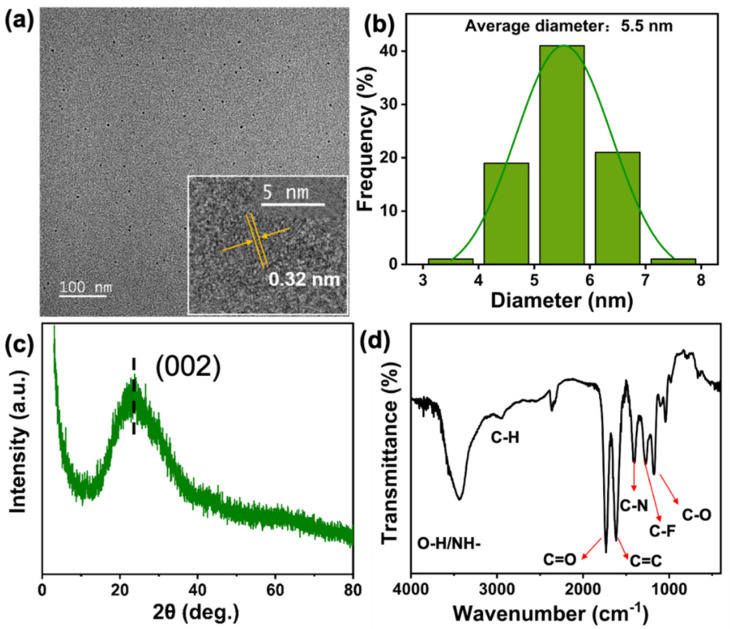
The structure characterization of NFCDs: (**a**) The well-dispersant quasi-spherical NFCDs particles were seen in high-resolution transmission electron microscope (HR-TEM) images. Inset: the lattice space of 0.32 nm. (**b**) The average diameter of NFCDs, determined by Gaussian fitting. (**c**) The 002 peak in XRD confirms the graphitic nature of the NFCD seen in the HR-TEM image (**a**). (**d**) The FT-IR spectrum of NFCDs presented the stretching vibrations of the surface functional groups.

In order to explore the morphology and structure of the NFCDs particles, the HR-TEM and XRD instruments were applied. The nanoparticles were found to be quasi-spherical (seen in Figure 1a), and the average size was estimated to be 5.5 ± 0.1 nm, based on 83 particles using the Gaussian distribution calculation (Figure 1b). The lattice spacing was further measured as 0.32 nm, corresponding to the (002) diffraction plane of graphite [43].

In addition, the morphology of the solid, detected by XRD in Figure 1c, demonstrated a broad peak centered at 23°, which was assigned to the disordered carbon in the NFCDs. In order to obtain a full understanding of the structure of NFCDs, the surface function groups were further detected by FT-IR spectroscopy, as seen in Figure 1d. A characteristic peak at around 3400 cm^−1^ of the FT-IR spectrum was observed, attributed to the stretching vibrations of the O-H and N-H functional groups. The peaks displayed in the region of 2850–2950 cm^−1^ were ascribed to the typical sp^3^ C-H stretching vibrations [44]. The peaks located at 1735, 1616, and 1404 cm^−1^ were related to the stretching vibrations of COOH, C=O, and C-N, respectively [40,45]. Moreover, the characteristic absorption peak of the C-F bond, together with the C=C stretching vibration, was observed at 1274 and 1175 cm^−1^ [40]. Thus, the as-prepared NFCDs possessed different functional groups, such as the hydroxyl, carboxyl, and amide groups, which are attributed to the fluorescence of the NFCDs [33].

**Figure 2:**
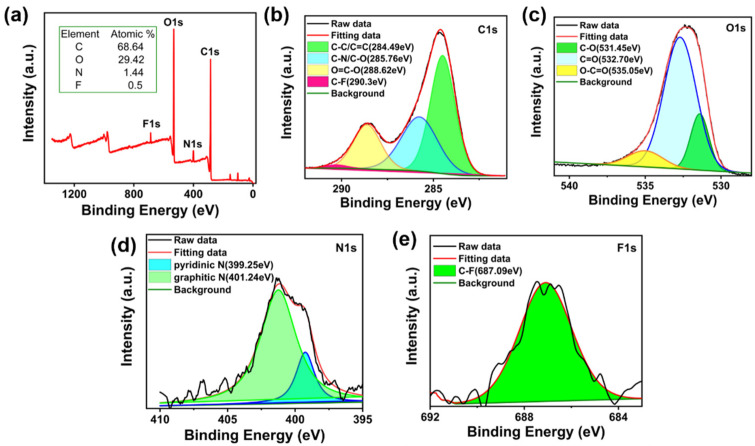
The XPS survey of NFCDs: (**a**) The full survey XPS spectrum of NFCDs presented the presence of C, N, O, and F elements. (**b**–**e**) The splitting of high-resolution C1s, O1s, N1s, and F1s confirmed the presence of surface functional groups observed in the FT-IR spectrum.

Furthermore, the XPS analysis was performed to estimate the chemical compositions. Figure 2a displayed the survey spectrum of the NFCDs, revealing four typical peaks at 284.7, 401.1, 532.3, and 687.6 eV. These binding energies were ascribed to C1s, N1s, O1s, and F1s, respectively [34,35]. The high-resolution spectra of C1s, as seen in Figure 2b, was deconvoluted into four typical peaks: C=C/C-C (284.49 eV) [45,46], C-N/C-O (285.76 eV) [17,47], O=C-O (288.62 eV) [35,48], and C-F (290.3 eV) [41]. Three peaks, corresponding to the binding energies of C-O (531.59 eV), C-O (532.97 eV), and O-C=O (534.16 eV), were obtained from the deconvolution of the O1s spectra, as shown in Figure 2c [29,35]. Apart from this, the splitting of the N1s peak in Figure 2d indicates the presence of pyridinic N (399.25 eV) and graphitic N (401.24 eV) [49,50]. Lastly, the F1s spectra displayed a single characteristic peak at 687.09 eV, assigned to the covalent C-F, respectively [40]. Thus, the XPS results of all the existing functional groups demonstrated a close match with the FT-IR analysis, confirming the presence of common luminescent centers [33].

### 3.2. Optical Property Characterization of NFCDs

**Figure 3:**
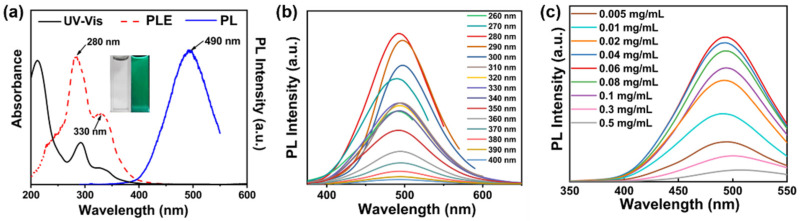
The optical properties of NFCDs solution at 0.02 mg/mL: (**a**) UV-vis absorption at 210 nm, 280 nm and 330 nm, and excitation peaks at 280 nm and 330 nm (PLE), together with 490 nm emission PL spectra (inset: left—NFCDs solution under daylight; right—NFCDs solution under UV light). (**b**) The emission spectra at 490 nm for NFCDs solution with different excitation wavelengths. (**c**) The emission spectra of NFCDs with concentrations ranging from 0.005 to 0.5 mg/mL.

The optical properties of the NFCDs solution were characterized. The photophysical properties of the as-synthesized NFCDs were examined through the ultraviolet-visible (UV-visible) and photoluminescence (PL) spectroscopies. As seen in Figure 3a, the UV-vis spectrum demonstrated three typical absorption bands. A strong absorption peak was observed at 210 nm, falling in the region of 210–250 nm, assigned to the aromatic π-π* transition of the C=C bond, while the second strong absorption peak, located at 290 nm, was related to the π-π* transitions of the C-N/C=N or C-F bonds [51,52]. Moreover, a distinct peak, observed at 330 nm, was attributed to the n-π* transitions of the C=O/C=N bonds in NFCDs [27,53]. The inset displayed a colorless solution under daylight and the one with an intense green fluorescence under UV light. Thus, the carbon cores and surface functional groups of the NFCDs absorbed the energies provided by UV light first, for which the excited state, in turn, drops to the ground state by emitting green fluorescence. Moreover, an optimal emission at around 490 nm (λ_ex_ = 280 nm) was displayed with the corresponding excitation spectra in Figure 3a, demonstrating two broad excitation peaks at 280 nm and 330 nm, respectively.

Figure 3b illustrates the PL spectra of the NFCDs solution upon different excitation wavelengths. The optimal emission spectra with a maximum intensity were obtained at around 490 nm under an excitation wavelength of 280 nm, which corresponded to the green fluorescence seen under the UV lamp. It was found that the emission spectra centered at 490 nm hardly shifted with the change of the excitation wavelengths (less than 10 nm), which might be a result of the monotonous surface defeats from the uniform preparation conditions and heteroatom dopant [3,38,44,54,55]. The QY was measured as 21.03%, which was higher than the QYs of most DL-MA-derived CDs [37,56,57].

Furthermore, the effect of concentration on the PL properties of the NFCDs was examined with different concentrations (0.005, 0.01, 0.02, 0.04, 0.06, 0.08, 0.1, 0.3, and 0.5 mg/mL). As presented in Figure 3c, the emission spectra were centered at 490 nm for all the concentrations considered, indicating a concentration-independent optical property. Interestingly, the PL intensity was enhanced when the concentration increased from 0.005 mg/mL until 0.06 mg/mL, after which it started decreasing from 0.06 to 0.5 mg/mL.

To further understand the PL behavior, the photoluminescence excitation (PLE) spectra of the solutions ranging from 0.005 to 0.5 mg/mL and the 3D spectra with concentrations of 0.04 mg/mL and 0.5 mg/mL were collected and displayed in Figure S1. Along with the increase in the NFCDs concentration from 0.005 mg/mL to 0.06 mg/mL, the intensities of the excitation peaks at around 280 nm and 330 nm were enhanced accordingly. At the higher concentrations from 0.08 mg/mL to 0.5 mg/mL, the excitation peaks at 280 and 330 nm gradually decreased, and an increase in the excitation centers at 360 nm was observed, indicating a change in the luminescent centers. The phenomenon might be that the n-π* transition of the aggregated hydroxyl groups absorbed 360 nm UV light, which contributes to the fluorescence in the concentrated solutions [34]. The intensity of the excitation peak at 280 nm became lower than that at 360 nm when the concentration was over 0.3 mg/mL. As NFCDs possess hydroxyl and carboxyl groups, at high concentrations, the surface functional groups are more likely to aggregate than that at smaller concentrations, thus leading to a slight change in the excitation peak heights. Therefore, the excitation peaks at 280 nm and 330nm became lower than that at 360 nm, resulting in a decrease in emission intensities [34,58].

### 3.3. Stability of NFCDs

The stability of the NFCDs was investigated under different experimental conditions, including varying UV light exposure times, ionic strengths, and pH values. As seen in Figure S2, the photostability of the NFCDs at 0.02 mg/mL was explored under continuous UV irradiation at 365 nm. After 120 min, the PL intensity remained comparable with that before any treatment, indicating an excellent photostability of NFCDs. Meanwhile, the NFCDs possessed almost 80% of the original PL intensity when the NaCl concentration reached 2 mol/L, presenting excellent stability with different ionic strengths, as seen in Figure S3.

**Figure 4:**
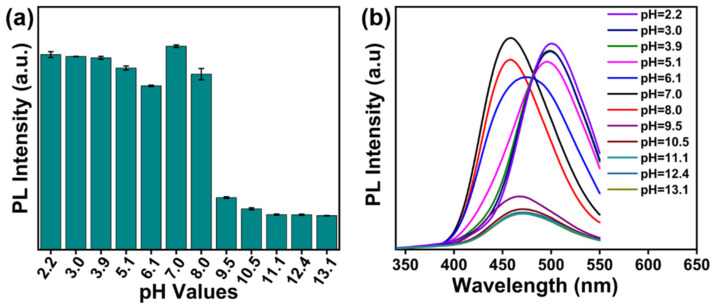
The effect of solution environments on PL spectra: (**a**) PL intensity decreases slightly in acid solution for pH from 2.2 to 6.1, and a sharp decrease in PL intensity in a basic environment. Error bars show standard deviations of repetitive experiments. (**b**) The slight red shift of PL spectra under both acid and basic environments.

Additionally, the optical properties of the NFCDs in different acidic and basic environments were also measured and displayed in Figure 4. The results illustrated that the PL intensity in an acidic medium decreased mildly when the pH values increased from 2.2 to 6.1, which reached a maximum intensity at 7.0. In the basic solutions, a sharp decrease in the PL intensities was observed for the pH values ranging from 8.0 to 13.1 as well as a blue shift of the emission peak from around 490 to 470 nm. The effects of the solution environments on the PL properties might be attributed to the protonation or deprotonation between the oxygen/nitrogen-containing groups and H^+^ or OH^−^ ions in the acidic or basic conditions, thus leading to a weaker fluorescence compared to the neutral environment [59,60].

### 3.4. Sensitivity and Selectivity Study of Fe^3+^ Ion

**Figure 5:**
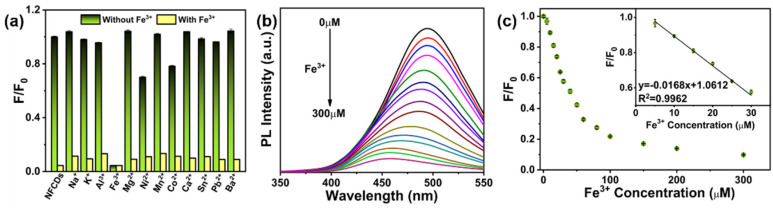
(**a**) The fluorescence quenching effect of different metal ions: dark green column-relative PL intensity with different metal ions; yellow column- relative PL intensity with different metal ions and Fe^3+^. (**b**) The PL spectra of NFCDs with various concentrations of Fe^3+^, ranging from 0 to 300 µM. (**c**) The relative PL intensity (F/F_0_) decay with various concentrations of Fe^3+^: inset: linear relationship between quenched intensity with concentrations of Fe^3+^, y = −0.0168x + 1.0612, R^2^ = 0.9962.

To assess the ability of the as-prepared NFCDs as a fluorescent probe for detecting metal ions, the fluorescence of the NFCDs at 0.02 mg/mL with various metal ions, including Na^+^, K^+^, Al^3+^, Fe^3+^, Mg^2+^, Ni^2+^, Mn^2+^, Co^2+^, Ca^2+^, Sn^2+^, Pb^2+^, and Ba^2+^, were measured and displayed in Figure 5a (as dark green columns, also seen in Table S1). The presence of Fe^3+^ showed the strongest quenching effects on the fluorescence, while the other ions presented limited quenching abilities. This phenomenon might be related to the intense affinity of the oxygen/nitrogen-contained groups for Fe^3+^, resulting in strong interactions between the NFCDs and Fe^3+^, thus leading to a quenched fluorescence [26]. Thus, the NFCDs can be utilized as a fluorescent probe for the detection of Fe^3+^ ions in a solution.

The sensitivity to detect Fe^3+^ with an NFCDs probe was evaluated with various Fe^3+^ concentrations from 5 µM to 300 µM in a 0.02 mg/mL NFCDs solution. As shown in Figure 5b, the PL fluorescence intensity was found to decrease sharply with the increased concentrations of Fe^3+^. While the concentration reached 300 µM, around 90% of the PL fluorescence was quenched, indicating a strong detection ability provided by the NFCDs. A blue shift of the emission spectra was observed with increasing Fe^3+^ concentrations, suggesting the formation of new fluorescence centers. The phenomenon might be explained by the chelation reaction between the Fe^3+^ and -COOH, -OH, and other groups on the surface of carbon cores [5]. Moreover, as shown in the inset of Figure 5c, a linear relationship between the ferric ion concentrations and PL intensities was obtained (R^2^ = 0.9962) when the concentration was from 5 to 30 µM. Additionally, the detection limit (LOD) was estimated to be 1.03 µM, which is comparable with the values obtained from the other systems (Table S2).

Furthermore, the selectivity of the NFCDs for Fe^3+^ was also investigated with various interference ions, including Na^+^, K^+^, Al^3+^, Mg^2+^, Ni^2+^, Mn^2+^, Co^2+^, Ca^2+^, Sn^2+^, Pb^2+^, and Ba^2+^. Figure 5a (yellow column) shows the PL intensity ratio (F/F_0_) with 500 µM interference ions with and without the addition of Fe^3+^. Furthermore, the presence of other metal ions had a negligible influence on the quenched fluorescence caused by Fe^3+^. Therefore, the NFCDs solution presented high sensitivity and excellent selectivity for Fe^3+^ detection.

### 3.5. Sensitivity and Selectivity Study of AA

**Figure 6:**
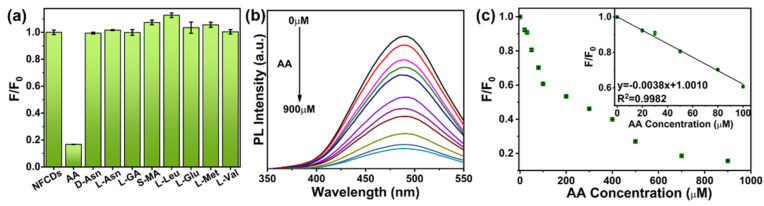
(**a**) The relative fluorescence intensity of NFCDs with the presence of different organic molecules. Error bars show standard deviations of repetitive experiments. (**b**) The PL spectra of NFCDs with various concentrations of AA, ranging from 0 to 900 µM. (**c**) The relative PL intensity (F/F_0_) decay with various concentrations of AA: inset: linear relationship between quenched intensity with concentrations of AA, y = −0.0038x + 1.0010, R^2^ = 0.9982.

The detection capabilities of the NFCDs were explored with the presence of different organic molecules (i.e., L-ascorbic acid (AA), D-asparagine (D-Asn), L-asparagine (L-Asn), L-glutamic acid (L-GA), S-mandelic acid (S-MA), L-leucine (L-Leu), L-glutamine (L-Glu), L-methionine (L-Met), and L-valine (L-Val)). As shown in Figure 6a and Table S3, the presence of organic molecules had almost no effect on the fluorescence of the NFCDs solution, except for the AA. It was observed that 500 µM of AA in 0.02 mg/mL NFCDs has caused an 83% reduction of the original PL intensity. The quenching phenomenon might be due to the complex interactions between the hydroxyl functional groups of AA with the hydrophilic functional groups of NFCDs [61].

To further explore the detection sensitivity of the NFCDs, the fluorescence spectra of NFCDs solutions with various concentrations of AA (0 to 900 µM) were examined and displayed in Figure 6b. As the AA concentration increased, the PL intensity at 490 nm decreased gradually, indicating increased quenching effects. A good linear relationship between the quenching ratio (F/F_0_) and AA concentration was exhibited from 5 to 100 µM (inset of Figure 6c), with the slope and LOD estimated as −0.0039 µM^−1^ and 4.22 µM, respectively. Thus, the NFCDs can be used as a “turn-off” sensor for AA detection, which is beneficial due to the easy sample preparation and one-step detection procedure. Table S4 presents the comparison of the current AA detection modes, linear ranges, and LODs from recent reports. Thus, the NFCDs prepared in the present work showed comparable LODs and linear ranges when compared with previous research [9,10,26,37].

### 3.6. Possible Quenching Mechanism of Fe^3+^ and AA

To further explore the quenching mechanism, the Stern–Volmer equation (Equation (1)) was used to model the relation between the F_0_/F versus Fe^3+^ and AA concentrations [62].

F_0_/F = 1 + k_sv_[Q], (1)

where F represents the PL intensity of the quenched solution; F_0_ is the original PL intensity without the presence of Fe^3+^; k_sv_ stands for the quenching constant in the Stern–Volmer equation; k_q_ presents the quenching constant, and τ is the PL lifetime of the NFCDs. As demonstrated in Figure S4a, the slope of the PL intensity ratio versus Fe^3+^ concentration was determined as 0.0318 µM^−1^, based on which the quenching rate constant was estimated to be 4.14 × 10^12^ M^−1^·s^−1^. Additionally, the Stern–Volmer equation, modeling the relation between the F_0_/F versus AA concentrations, was displayed in Figure S6a, with the quenching rate constant, k_q_, determined as 7.72 × 10^11^ M^−1^·s^−1^. Both the quenching constants for the Fe^3+^ and AA were one or two orders of magnitude higher than the general considered dynamic quenching constant (DQM), 1.0 × 10^10^ M^−1^·s^−1^. Thus, the static quenching mechanism (SQM), referring to the reactions between the NFCDs and Fe^3+^ or AA, might be responsible for the quenching phenomenon caused by Fe^3+^ and AA [63,64].

This assumption was further supported by the UV-vis spectra and PL lifetime analysis. Figure S4b shows the UV absorption curves of the NFCDs and NFCDs/Fe^3+^ solutions. The absorption band of the NFCDs at 330 nm was gradually reduced along with the addition of Fe^3+^, confirming the formation of a non-fluorescent NFCDs-Fe^3+^ chelating complex (SQM). Additional evidence was supplied by the PL lifetime analysis. The fluorescence lifetime of the NFCDs with 500 µM Fe^3+^ was estimated as 8.29 ns (Figure S5a), showing a slight increase rather than a decrease compared to 7.69 ns for the NFCDs. Thus, it is reasonable to eliminate the possibility of DQM [25]. Moreover, Figure S5b displays the UV-vis spectrum of the Fe^3+^ solution as well as the excitation and emission spectra of the NFCDs. A broad absorption band at 280 nm was seen in the UV-vis spectrum, which overlaps with the excitation peaks of the NFCDs at 280 nm. Thus, the possible quenching mechanism of Fe^3+^ against the NFCDs might be a combination of the inner filter effect (IFE) and SQE [65]. For the NFCDs/AA system, Figure S6b shows the UV-vis spectra of the NFCDs and NFCDs/AA solutions, where an absorption peak centered at 250 nm appeared and became stronger with an increased AA concentration. This observation confirmed the formation of the NFCDs/AA complex, which contributed to the quenched fluorescence of NFCDs and thus supported SQM.

**Figure 7:**
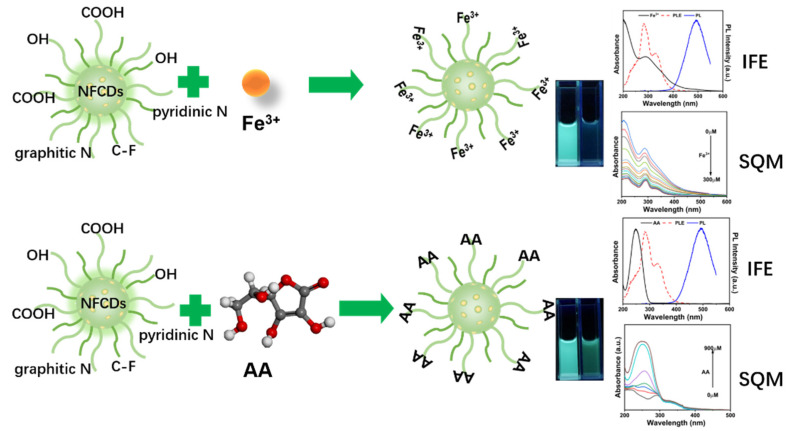
Possible quenching mechanism of NFCDs of Fe^3+^ and AA: (**Top**) the addition of Fe^3+^ with different concentrations interacts with the surface functional groups to form a non-fluorescent complex (SQM); UV-vis spectra show overlaps from 280 to 330 nm between UV absorption (black line) and PLE of NFCDs (red line). (**Bottom**) the addition of AA interacts with the surface functional groups to form a non-fluorescent complex (SQM); UV-vis spectra show overlaps from 200 to 300 nm between UV absorption (black line) and PLE of NFCDs (red line).

Furthermore, the UV-vis spectrum of AA is supplied in Figure S7b, together with the excitation and emission fluorescence spectra. The characteristic absorption of AA was in a range from 210 to 300 nm, overlapping with the excitation centers of NFCDs. Thus, the AA might shield the excitation light of the NFCDs, resulting in decreased PL intensities. Additionally, the PL decay measurements illustrated that the lifetime for the NFCDs/AA solution (8.04 ns, as seen in Figure S7a) showed a negligible difference from the NFCDs’ lifetime. Thus, an inner filter effect (IFE) was presented and contributed to the quenching effects in the presence of AA. Therefore, NFCDs can be potentially used as a “turn-off” sensor for the detection of AA based on the SQM and IFE processes. The possible quenching mechanism for both Fe^3+^ and AA is presented in Figure 7, providing different states of NFCDs particles and the corresponding UV-vis spectra with the excitation and emission spectra. Thus, both the IFE and SQM contributed to the quenching phenomenon of NFCDs with the presence of Fe^3+^ and AA, respectively.

## 4. Conclusions

In conclusion, a fluorescent carbon-based probe with a high quantum yield of 21.03% was directly developed using DL-malic acid and levofloxacin through a rapid and low-cost microwave treatment. The as-prepared NFCDs exhibited excellent water dispersibility with an average size of 5.49 nm. Additionally, the excitation-independent and concentration-independent fluorescence behaviors of the NFCDs were observed with a single emission of 490 nm. Moreover, the green fluorescence can be quenched by both Fe^3+^ and AA as “turn-off” probes, with LODs of 1.03 and 4.22 µM. The quenching mechanisms were further determined as a static quenching mechanism and inner filter effect, based on the lifetime and spectral analysis. This methodology provided a rapid and low-cost approach to the synthesis of nitrogen and fluorine co-doped CDs with a high quantum yield, rapid response, and high sensitivity and selectivity as fluorescent probes to detect Fe^3+^ and AA in biological and environmental systems.

## Data Availability

The data presented in this study are available on request from the corresponding author.

## Supplementary Materials

The following supporting information can be downloaded at: https://www.mdpi.com/article/10.3390/nano12142377/s1, Figure S1: (a,b) The normalized excitation peaks under emission peaks at 490 nm with various concentrations of NFCDs, (c,d) 3D fluorescent matrix scan of the NFCDs at 0.04 mg/mL and 0.5 mg/mL; Figure S2: Relationship between F/F_0_ and the irradiation time, where F_0_ and F are the emission intensity before and after UV light irradiation, respectively; Figure S3: Relationship between F/F_0_ and the ionic strength, where F_0_ and F are the emission intensity in ultrapure water and NaCl solution, respectively; Figure S4: (a) Stern–Volmer relationship between F_0_/F and concentration of Fe^3+^, (b) UV-vis spectra of NFCDs solution with different concentrations of Fe^3+^; Figure S5: (a) The PL decay lifetime of NFCDs solution with and without the presence of Fe^3+^, (b) UV-vis spectra of Fe^3+^ solution, the emission peaks excited at 280 nm and the excitation peak at the emission of 490 nm of NFCDs solution; Figure S6: (a) Stern–Volmer relationship between F_0_/F and concentration of AA, (b) UV-vis spectra of NFCDs solution with different concentrations of AA; Figure S7: (a) The PL decay lifetime of NFCDs solution with and without the presence of AA, (b) UV-vis spectra of Fe^3+^ solution, the emission peaks excited at 280 nm and the excitation peak at the emission of 490 nm of NFCDs solution; Table S1: Selectivity and sensitivity results of NFCDs for the detection of metal ions; Table S2: Comparison of different probes and methods for detection of Fe^3+^; Table S3: sensitivity test of NFCDs with the presence of different organic molecules; Table S4: Comparison of different probes and methods for detection of AA [66,67,68,69,70].

## References

[1] Andreani A.S., Kunarti E.S., Hashimoto T., Hayashita T., Santosa S.J. (2021). Fast and selective colorimetric detection of Fe^3+^ based on gold nanoparticles capped with ortho-hydroxybenzoic acid. J. Environ. Chem. Eng..

[2] Cheng H.Y., Li D.C., Cheng B.H., Jiang H. (2019). Highly stable and selective measurement of Fe^3+^ ions under environmentally relevant conditions via an excitation-based multiwavelength method using N, S-doped carbon dots. Environ. Res..

[3] Lesani P., Ardekani S.M., Dehghani A., Hassan M., Gomes V.G. (2019). Excitation-independent carbon dot probes for exogenous and endogenous Fe^3+^ sensing in living cells: Fluorescence lifetime and sensing mechanism. Sens. Actuators B Chem..

[4] Nagaraj M., Ramalingam S., Murugan C., Aldawood S., Jin J.O., Choi I., Kim M. (2022). Detection of Fe^3+^ ions in aqueous environment using fluorescent carbon quantum dots synthesized from endosperm of Borassus flabellifer. Environ. Res..

[5] Liu Z., Jia R., Chen F., Yan G., Tian W., Zhang J., Zhang J. (2022). Electrochemical process of early-stage corrosion detection based on N-doped carbon dots with superior Fe^3+^ responsiveness. J. Colloid Interface Sci..

[6] Eisenstein R.S. (2000). Iron regulatory proteins and the molecular control of mammalian iron metabolism. Ann. Rev. Nutr..

[7] Cairo G., Pietrangelo A. (2000). Iron regulatory proteins in pathobiology. Biochem. J..

[8] Bogireddy N.K.R., Sotelo Rios S.E., Agarwal V. (2021). Simple one step synthesis of dual-emissive heteroatom doped carbon dots for acetone sensing in commercial products and Cr (VI) reduction. Chem. Eng. J..

[9] Shi H., Chen L., Niu N. (2021). An off-on fluorescent probe based on graphene quantum dots intercalated hydrotalcite for determination of ascorbic acid and phytase. Sens. Actuators B Chem..

[10] Wang T., Luo H., Jing X., Yang J., Huo M., Wang Y. (2021). Synthesis of Fluorescent Carbon Dots and Their Application in Ascorbic Acid Detection. Molecules.

[11] Zhang W., Wang R.X., Li P., Zhang W., Pang X., Wang H., Tang B. (2019). Fluorescence biosensor for Fe(III) in cells based on Fe(III) catalytze Au-nanocomposites release Au NPs. Sens. Actuators B.

[12] Lin X., Xuan D.L., Li F.F., Liu C., Fan P.F., Xiao F.B., Liang H., Yang S.Y. (2020). DNA-AgNCs as a fluorescence turn-off probe for dual functional detection of H_2_O_2_ and Fe(II) ions. Spectrochim. Acta A.

[13] Zhu D., Xue L., Li G., Jiang H. (2016). A highly sensitive near-infrared ratiometric fluorescent probe for detecting nitroreductase and cellular imaging. Sens. Actuators B.

[14] Lu Y., Yan B., Liu J.L. (2014). Nanoscale metal-organic frameworks as highly sensitive luminescent sensors for Fe^2+^ in aqueous solution and living cells. Chem. Commun..

[15] Wu P., Hou X., Xu J.-J., Chen H.-Y. (2016). Ratiometric fluorescence, electrochemiluminescence, and photoelectrochemical chemo/biosensing based on semiconductor quantum dots. Nanoscale.

[16] Huang Z.Z., Song W.Z., Li Y., Wang L.Y., Pandey N.K., Chudal L., Wang M., Li Y.C., Zhao L.L., Yin W.Z. (2020). The exploration of novel fluorescent copper-cysteamine nanosheets for sequential detection of Fe^3+^ and dopamine and fabrication of molecular logic circuits. J. Mater. Chem. C.

[17] Zhang Y., Qin H., Huang Y., Zhang F., Liu H., Liu H., Wang Z.J., Li R. (2021). Highly fluorescent nitrogen and boron doped carbon quantum dots for selective and sensitive detection of Fe^3+^. J. Mater. Chem. B.

[18] Das P., Ganguly S., Margel S., Gedanken A. (2021). Immobilization of Heteroatom-Doped Carbon Dots onto Nonpolar Plastics for Antifogging, Antioxidant, and Food Monitoring Applications. Langmuir.

[19] Fu Y., Zhao S., Wu S., Huang L., Xu T., Xing X., Lan M., Song X. (2020). A carbon dots-based fluorescent probe for turn-on sensing of ampicillin. Dye. Pigment..

[20] Das P., Bose M., Das A.K., Banerjee S., Das N.C. (2018). One-Step Synthesis of Fluorescent Carbon Dots for Bio-Labeling Assay. Macromol. Symp..

[21] Sharma V., Tiwari P., Mobin S.M. (2017). Sustainable carbon-dots: Recent advances in green carbon dots for sensing and bioimaging. J. Mater. Chem. B.

[22] Xu X., Ren D., Chai Y., Cheng X., Mei J., Bao J., Wei F., Xu G., Hu Q., Cen Y. (2019). Dual-emission carbon dots-based fluorescent probe for ratiometric sensing of Fe(III) and pyrophosphate in biological samples. Sens. Actuators B Chem..

[23] Gong X., Lu W., Paau M.C., Hu Q., Wu X., Shuang S., Dong C., Choi M.M.F. (2015). Facile synthesis of nitrogen-doped carbon dots for Fe^3+^ sensing and cellular imaging. Anal. Chim. Acta..

[24] Wang Y., He Q., Zhao X., Yuan J., Zhao H., Wang G., Li M. (2022). Synthesis of corn straw-based graphene quantum dots (GQDs) and their application in PO_4_^3−^ detection. J. Environ. Chem. Eng..

[25] Shen C., Dong C., Cheng L., Shi X., Bi H. (2022). Fluorescent carbon dots from *Shewanella oneidensis* MR–1 for Hg^2+^ and tetracycline detection and selective fluorescence imaging of Gram–positive bacteria. J. Environ. Chem. Eng..

[26] Li C., Zeng J., Guo D., Liu L., Xiong L., Luo X., Hu Z., Wu F. (2021). Cobalt-Doped Carbon Quantum Dots with Peroxidase-Mimetic Activity for Ascorbic Acid Detection through Both Fluorometric and Colorimetric Methods. ACS Appl. Mater. Interfaces.

[27] Xu M.S., Dong C., Xu J.H., Rehman S.U., Wang Q.Y., Osipov V.Y., Jiang K., Wang J.F., Bi H. (2022). Fluorinated carbon dots/carboxyl methyl cellulose sodium composite with a temperature-sensitive fluorescence/phosphorescence applicable for anti-counterfeiting marking. Carbon.

[28] Li X., Fu Y., Zhao S., Xiao J., Lan M., Wang B., Zhang K., Song X., Zeng L. (2022). Metal ions-doped carbon dots: Synthesis, properties, and applications. Chem. Eng. J..

[29] Wang J., Li Q., Zheng J., Yang Y., Liu X., Xu B. (2021). N, B-Codoping Induces High-Efficiency Solid-State Fluorescence and Dual Emission of Yellow/Orange Carbon Dots. ACS Sustain. Chem. Eng..

[30] Fu Q., Long C., Huang J., Liu S., Qing T., Zhang P., Feng B. (2021). Highly sensitive B, N co-doped carbon dots for fluorescent and colorimetric dual-mode detection of mercury ions in wastewater. J. Environ. Chem. Eng..

[31] Ge J., Shen Y., Wang W., Li Y., Yang Y. (2021). N-doped carbon dots for highly sensitive and selective sensing of copper ion and sulfide anion in lake water. J. Environ. Chem. Eng..

[32] Wang W., Peng J., Li F., Su B., Chen X., Chen X. (2018). Phosphorus and chlorine co-doped carbon dots with strong photoluminescence as a fluorescent probe for ferric ions. Microchim. Acta.

[33] Sun Z., Zhou W., Luo J., Fan J., Wu Z.-c., Zhu H., Huang J., Zhang X. (2022). High-efficient and pH-sensitive orange luminescence from silicon-doped carbon dots for information encryption and bio-imaging. J. Colloid Interface Sci..

[34] Bai L., Yan H., Feng Y., Feng W., Yuan L. (2019). Multi-excitation and single color emission carbon dots doped with silicon and nitrogen: Synthesis, emission mechanism, Fe^3+^ probe and cell imaging. Chem. Eng. J..

[35] Liu F., Li Z., Li Y., Feng Y., Feng W. (2021). Room-temperature phosphorescent fluorine-nitrogen co-doped carbon dots: Information encryption and anti-counterfeiting. Carbon.

[36] Liu Q., Niu X., Xie K., Yan Y., Ren B., Liu R., Li Y., Li L. (2021). Fluorescent Carbon Dots as Nanosensors for Monitoring and Imaging Fe^3+^ and [HPO_4_]^2–^ Ions. ACS Appl. Nano Mater..

[37] Cui J., Zhu X., Liu Y., Liang L., Peng Y., Wu S., Zhao Y. (2022). N-Doped Carbon Dots as Fluorescent “Turn-Off” Nanosensors for Ascorbic Acid and Fe^3+^ Detection. ACS Appl. Nano Mater..

[38] Mishra L., Behera R.K., Mondal S., Kumar S., Panigrahi A., Sarangi M.K. (2021). Interface and doping in carbon dots influence charge transfer and transport. Carbon.

[39] Zhu J., Chu H., Shen J., Wang C., Wei Y. (2021). Nitrogen and fluorine co-doped green fluorescence carbon dots as a label-free probe for determination of cytochrome c in serum and temperature sensing. J. Colloid Interface Sci..

[40] Wu X., Xu M., Wang S., Abbas K., Huang X., Zhang R., Tedesco A.C., Bi H.F. (2022). N-Doped carbon dots as efficient Type I photosensitizers for photodynamic therapy. Dalton Trans..

[41] Ding H., Xu J., Jiang L., Dong C., Meng Q., Rehman S.u., Wang J., Ge Z., Osipov V.Y., Bi H. (2021). Fluorine-defects induced solid-state red emission of carbon dots with an excellent thermosensitivity. Chin. Chem. Lett..

[42] Wang C., Chen D., Yang Y., Tang S., Li X., Xie F., Wang G., Guo Q. (2021). Synthesis of multi-color fluorine and nitrogen co-doped graphene quantum dots for use in tetracycline detection, colorful solid fluorescent ink, and film. J. Colloid Interface Sci..

[43] Mo L., Xu X., Liu Z., Liu H., Lei B., Zhuang J., Guo Z., Liu Y., Hu C. (2021). Visible-light excitable thermally activated delayed fluorescence in aqueous solution from F, N-doped carbon dots confined in silica nanoparticles. Chem. Eng. J..

[44] Zhu L., Shen D., Wang Q., Luo K.H. (2021). Green Synthesis of Tunable Fluorescent Carbon Quantum Dots from Lignin and Their Application in Anti-Counterfeit Printing. ACS Appl. Mater. Interfaces.

[45] Shen J., Xu B., Wang Z., Zhang J., Zhang W., Gao Z., Wang X., Zhu C., Meng X. (2021). Aggregation-induced room temperature phosphorescent carbonized polymer dots with wide-range tunable lifetimes for optical multiplexing. J. Mater. Chem. C.

[46] Zhu P., Li J., Gao L., Xiong J., Tan K. (2021). Strategy to Synthesize Tunable Multiemission Carbon Dots and Their Multicolor Visualization Application. ACS Appl. Mater. Interfaces.

[47] Tan J., Li Q., Meng S., Li Y., Yang J., Ye Y., Tang Z., Qu S., Ren X. (2021). Time-Dependent Phosphorescence Colors from Carbon Dots for Advanced Dynamic Information Encryption. Adv. Mater..

[48] Long P., Feng Y.Y., Cao C., Li Y., Han J.K., Li S.W., Peng C., Li Z.Y., Feng W. (2018). Self-Protective Room-Temperature Phosphorescence of Fluorine and Nitrogen Codoped Carbon Dots. Adv. Funct. Mater..

[49] Sim Y., Kim S.J., Janani G., Chae Y., Surendran S., Kim H., Yoo S., Seok D.C., Jung Y.H., Jeon C. (2020). The synergistic effect of nitrogen and fluorine co-doping in graphene quantum dot catalysts for full water splitting and supercapacitor. Appl. Surf. Sci..

[50] Liu E., Li D., Zhou X., Zhou G., Xiao H., Zhou D., Tian P., Guo R., Qu S. (2019). Highly Emissive Carbon Dots in Solid State and Their Applications in Light-Emitting Devices and Visible Light Communication. ACS Sustain. Chem. Eng..

[51] Wang B., Mu Y., Zhang C., Li J. (2017). Blue photoluminescent carbon nanodots prepared from zeolite as efficient sensors for picric acid detection. Sens. Actuators B Chem..

[52] Ma W., Wang B., Yang Y., Li J. (2021). Photoluminescent chiral carbon dots derived from glutamine. Chin. Chem. Lett..

[53] Macairan J.-R., de Medeiros T.V., Gazzetto M., Yarur Villanueva F., Cannizzo A., Naccache R. (2022). Elucidating the mechanism of dual-fluorescence in carbon dots. J. Colloid Interface Sci..

[54] Zheng Y., Arkin K., Hao J., Zhang S., Guan W., Wang L., Guo Y., Shang Q. (2021). Multicolor Carbon Dots Prepared by Single-Factor Control of Graphitization and Surface Oxidation for High-Quality White Light-Emitting Diodes. Adv. Opt. Mater..

[55] Chen Y., Lian H., Wei Y., He X., Chen Y., Wang B., Zeng Q., Lin J. (2018). Concentration-induced multi-colored emissions in carbon dots: Origination from triple fluorescent centers. Nanoscale.

[56] Hao X., Dai S., Wang J., Fang Z. (2021). Synthesis of blue fluorescent carbon dots and their application in detecting mercury and iodine based on “off–on” mode. Luminescence.

[57] Kang J.W., Kang D.H. (2021). Effect of amino acid-derived nitrogen and/or sulfur doping on the visible-light-driven antimicrobial activity of carbon quantum dots: A comparative study. Chem. Eng. J..

[58] Das P., Maruthapandi M., Saravanan A., Natan M., Jacobi G., Banin E., Gedanken A. (2020). Carbon Dots for Heavy-Metal Sensing, pH-Sensitive Cargo Delivery, and Antibacterial Applications. ACS Appl. Nano Mater..

[59] Pang S., Liu S. (2020). Dual-emission carbon dots for ratiometric detection of Fe^3+^ ions and acid phosphatase. Anal. Chim. Acta.

[60] Kalaiyarasan G., Joseph J., Kumar P. (2020). Phosphorus-Doped Carbon Quantum Dots as Fluorometric Probes for Iron Detection. ACS Omega.

[61] Liu Y., Wu P., Wu X., Ma C., Luo S., Xu M., Li W., Liu S. (2020). Nitrogen and copper (II) co-doped carbon dots for applications in ascorbic acid determination by non-oxidation reduction strategy and cellular imaging. Talanta.

[62] Yue J., Li L., Cao L., Zan M., Yang D., Wang Z., Chang Z., Mei Q., Miao P., Dong W.-F. (2019). Two-Step Hydrothermal Preparation of Carbon Dots for Calcium Ion Detection. ACS Appl. Mater. Interfaces.

[63] Hu G., Ge L., Li Y., Mukhtar M., Shen B., Yang D., Li J. (2020). Carbon dots derived from flax straw for highly sensitive and selective detections of cobalt, chromium, and ascorbic acid. J. Colloid Interface Sci..

[64] Long C., Jiang Z., Shangguan J., Qing T., Zhang P., Feng B. (2021). Applications of carbon dots in environmental pollution control: A review. Chem. Eng. J..

[65] Lin M., Zou H.Y., Yang T., Liu Z.X., Liu H., Huang C.Z. (2016). An inner filter effect based sensor of tetracycline hydrochloride as developed by loading photoluminescent carbon nanodots in the electrospun nanofibers. Nanoscale.

[66] Yu J., Xu C., Tian Z., Lin Y., Shi Z. (2016). Facilely synthesized N-doped carbon quantum dots with high fluorescent yield for sensing Fe^3+^. New J. Chem..

[67] Song Y., Zhu C., Song J., Li H., Du D., Lin Y. (2017). Drug-Derived Bright and Color-Tunable N-Doped Carbon Dots for Cell Imaging and Sensitive Detection of Fe^3+^ in Living Cells. ACS Appl. Mater. Interfaces.

[68] Zhao P., Zhang Q., Cao J., Qian C., Ye J., Xu S., Zhang Y., Li Y. (2022). Facile and Green Synthesis of Highly Fluorescent Carbon Quantum Dots from Water Hyacinth for the Detection of Ferric Iron and Cellular Imaging. Nanomaterials.

[69] Ye S., Zhang M., Guo J., Song J., Zeng P., Qu J., Chen Y., Li H. (2022). Facile Synthesis of Green Fluorescent Carbon Dots and Their Application to Fe^3+^ Detection in Aqueous Solutions. Nanomaterials.

[70] Gan L., Su Q., Chen Z., Yang X. (2020). Exploration of pH-responsive carbon dots for detecting nitrite and ascorbic acid. Appl. Surf. Sci..

