# Supplementary material for: One-Step Synthesis of Nitrogen/Fluorine Co-Doped Carbon Dots for Use in Ferric Ions and Ascorbic Acid Detection
Source: Nanomaterials (Basel). 2022 Jul 12;12(14):2377. doi: 10.3390/nano12142377 (PMC9323265; doi:10.3390/nano12142377)
Supplement: Supplementary file 1 [file nanomaterials-12-02377-s001.zip › nanomaterials-1806032-supplementary.pdf]

Supporting Information

One-Step Synthesis of Nitrogen/Fluorine Co-Doped Carbon Dots for Use in Ferric Ions and Ascorbic Acid Detection

Yan Zhao [1,*], Xiaoxuan Zhu [1], Lu Liu [1], Zhiqing Duan [1], Yanping Liu [1,2], Weiyuan Zhang [1], Jingjing Cui [1], Yafang Rong [3] and Chen Dong [4,*]

[1] School of Chemical Engineering, Hebei Normal University of Science and Technology, Qinhuangdao 066004, China; zhuxiaoxuan1999@163.com (X.Z.); 13633336800@163.com (L.L.); qizixuanyuanke@163.com (Z.D.); lyp3893@hevttc.edu.cn (Y.L.); 13649502263@163.com (W.Z.); cuijingjing23@outlook.com (J.C.)

[2] Hebei Key Laboratory of Active Components and Functions in Natural Product, Qinhuangdao 066004, China

[3] Shandong Zhengyuan Geophysical Information Technology Co., Ltd., Jinan 250000, China; 13953173230@139.com

[4] Cixi Institute of Biomedical Engineering, International Cooperation Base of Biomedical Materials Technology and Application, CAS Key Laboratory of Magnetic Materials and Devices, Zhejiang Engineering Research Center for Biomedical Materials, Ningbo Institute of Materials Technology and Engineering, Chinese Academy of Sciences, Ningbo 315201, China

* Correspondence: zy3780@hevttc.edu.cn (Y.Z.); dongchen1012@163.com (C.D.)

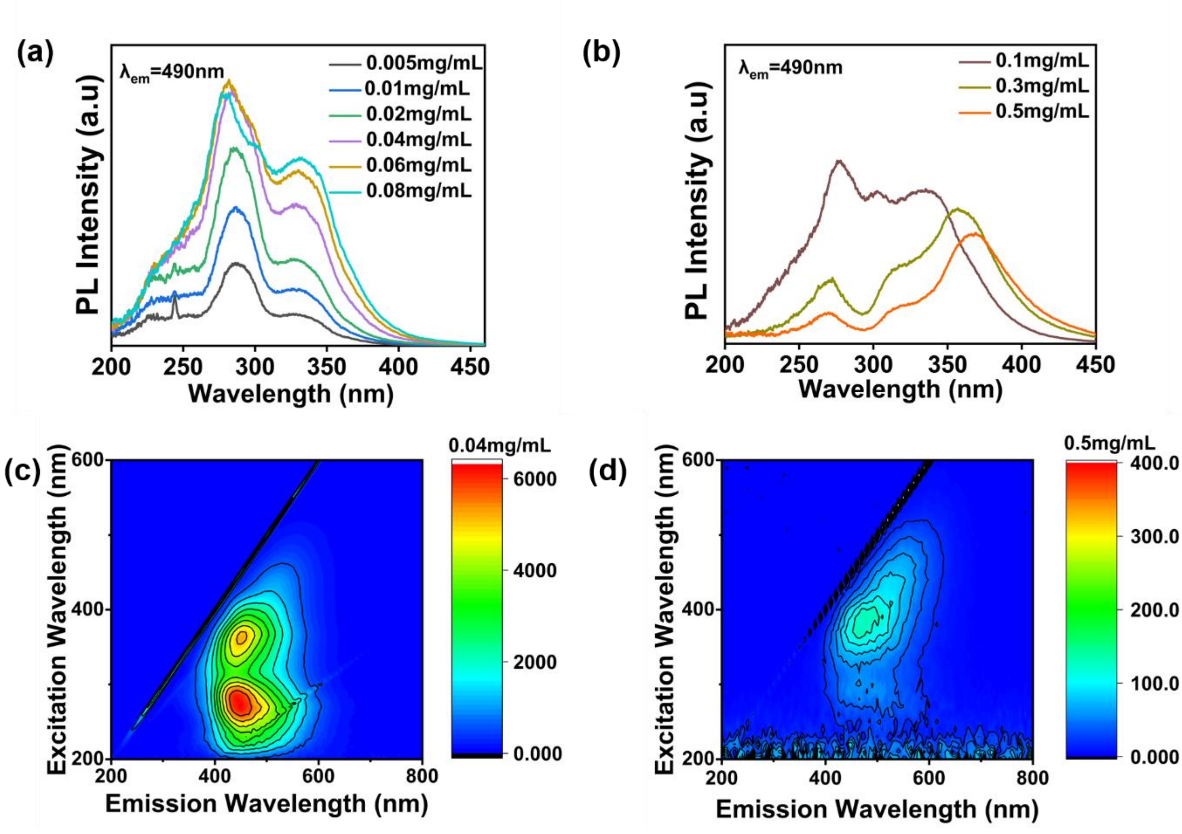

Figure S1. **(a)-(b)** The normalized excitation peaks under emission peaks at 490 nm with various concentrations of NCDs. **(c)-(d)** 3D fluorescent matrix scan of the NFCDs at 0.04 mg/mL and 0.5 mg/mL.

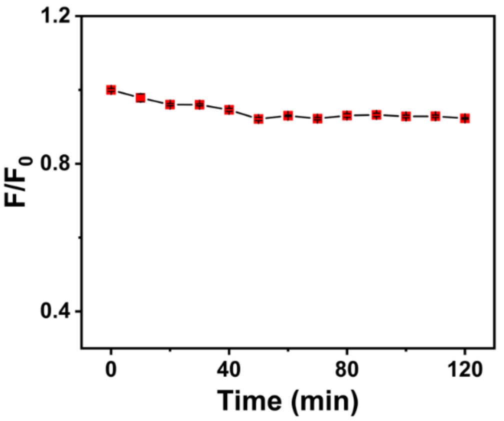

Figure S2. Relationship between F/F_0 and the irradiation time, where F_0 and F are the emission intensity before and after UV light irradiation, respectively.

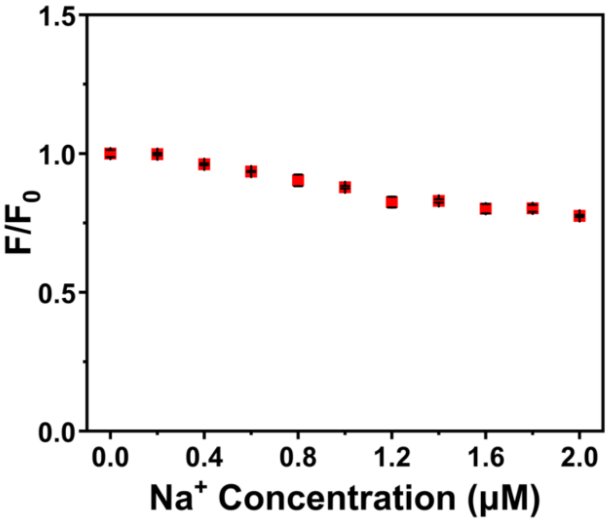

Figure S3. Relationship between F/F_0 and the ionic strength, where F_0 and F are the emission intensity in ultrapure water and NaCl solution, respectively.

Table S1 Selectivity and sensitivity results of NFCDs for the detection of metal ions

	F/F_0 without Fe^{3+}	Error bar	F/F0 with Fe^{3+}	Error bar
NFCDs	1.0000	0.0060	0.0448	0.0010
Na^{+}	1.0376	0.0107	0.1139	0.0009
K^{+}	0.9808	0.0045	0.0942	0.0017
Al^{3+}	0.9562	0.0043	0.1330	0.0032
Fe^{3+}	0.0448	0.0010	0.0448	0.0010
Mg^{2+}	1.0417	0.0104	0.0913	0.0026
Ni^{2+}	0.7002	0.0069	0.1104	0.0009
Mn^{2+}	1.0195	0.0077	0.1338	0.0005
Co^{2+}	0.7822	0.0060	0.1133	0.0007
Ca^{2+}	1.0378	0.0023	0.0999	0.0015
Sn^{2+}	0.9850	0.0083	0.1113	0.0012
Pb^{2+}	0.9627	0.0022	0.0898	0.0010
Ba^{2+}	1.0431	0.0161	0.0901	0.0010

Table S2. Comparison of different probes and methods for detection of Fe^{3+}

Fluorescent probe	Detection mode	Linear range/μM	LOD/μM	QY	Ref.
Borassus flabellifer derived CDs	turn-off	10-100	2.01	19.4%	[1]
L-glutamic carbon quantum dots	turn-off	0-50	4.67	17.8%	[2]
N-doped CDs	turn-off	5-60	1.9	9.8%	[3]
N-doped CDs	turn-off	10-300	0.9	-	[4]
Graphite electrode CDs	turn-off	10-200	1.8	16.4%	[5]
water hyacinth derived CDs	turn-off	0-330	0.084	3.3%	[6]
FNCDs	turn-off	0.2-300	0.08	-	[7]
1,2,4-triaminobenzene and formamide	turn-off	1-60	0.28	-	[8]
N-doped CDs	turn-off	0.001-1000	1.68	-	[9]
N,F co-doped CDs	turn-off	5-30	1.03	21.03%	this work

Table S3. sensitivity test of NFCDs with the presence of different organic molecules

F/F_0	R1	R2	R3	mean values
FNCDs	0.9876	0.9926	1.0198	1.0000
LGA	1.0230	0.9904	0.9830	0.9988
D-Asn	1.0011	0.9950	0.9869	0.9943
L-Asn	1.0166	1.0198	1.0127	1.0164
L-Glu	1.0807	1.0255	0.9993	1.0352
S-MA	1.0910	1.0733	1.0570	1.0738
L-Met	1.0407	1.0503	1.0765	1.0558
L-Leu	1.1416	1.1080	1.1317	1.1271
L-Val	1.0230	1.0000	0.9901	1.0044
AA	0.1687	0.1664	0.1681	0.1677

Table S4. Comparison of different probes and methods for detection of AA

Fluorescent probe	Detection mode	Linear range/μM	LOD/μM	QY	Ref.
CDs/Cr^{6+}	on-off-on	0-200	0.35	11.83%	[10]
Co-CDs/Fe^{3+}	on-off-on	0.6-1600	18	30.4%	[11]
Co-CDs/TMB	on-off-on	10-400	0.27	30.4%	[11]
CDs	on-off-on	0.1-800	50	20.7%	[12]
N, Cu-CDs	turn-off	0.02-40	0.018	-	[13]
N-CDs	turn-off	20-60	2.6	9.8%	[3]
N,F-CDs	turn-off	5-100	4.22	21.03%	this work

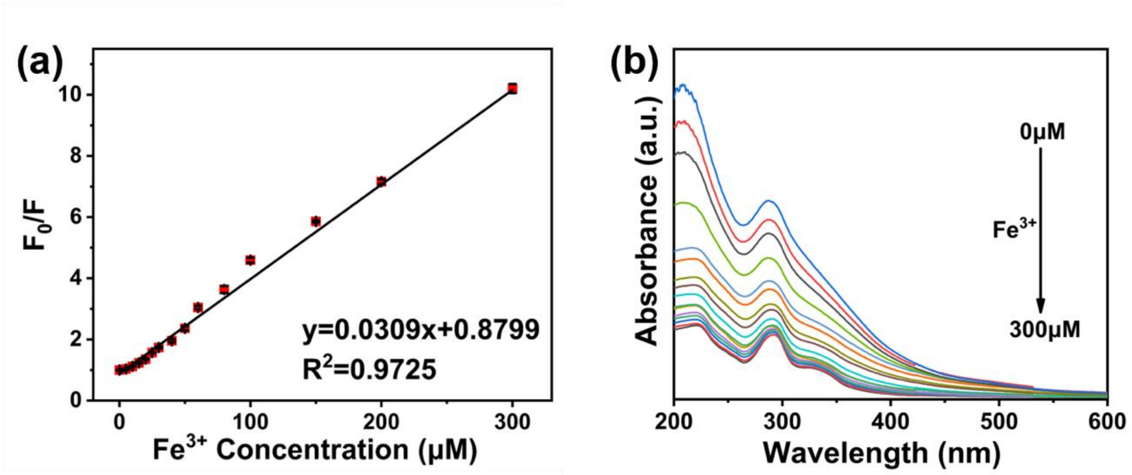

Figure S4. **(a)** Stern-Volmer relationship between F_0/F and concentration of Fe^{3+}. **(b)** UV-vis spectra of NFCDs solution with different concentration of Fe^{3+}.

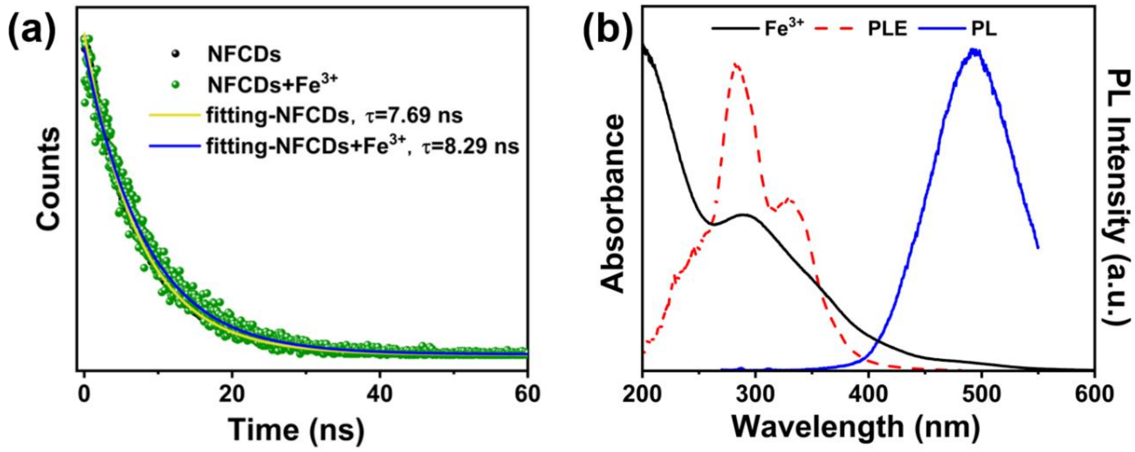

Figure S5. (a) The PL decay lifetime of NFCDs solution with and without the presence of Fe^{3+}. **(b)** UV-vis spectra of Fe^{3+} solution, the emission peaks excited at 280nm and the excitation peak at emission of 490 nm of NFCDs solution.

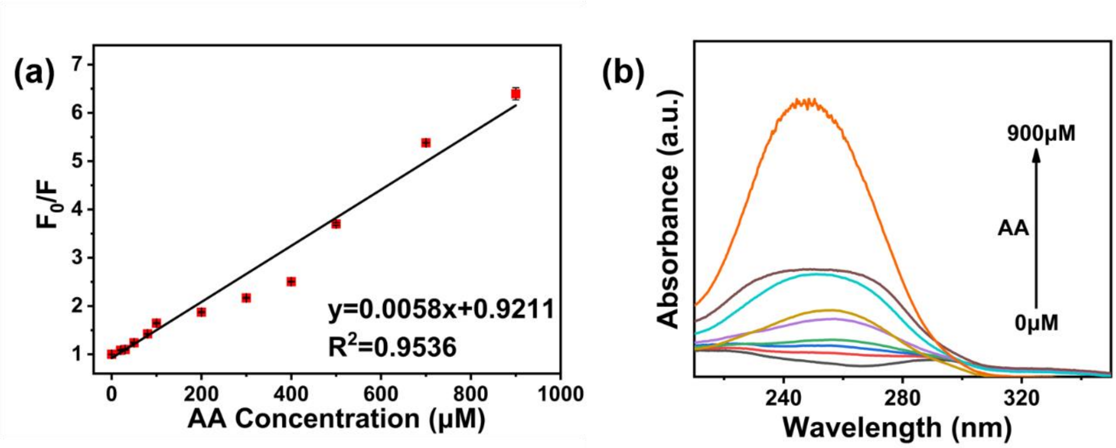

Figure S6. (a) Stern-Volmer relationship between F_0/F and concentration of AA. **(b)** UV-vis spectra of NFCDs solution with different concentration of AA.

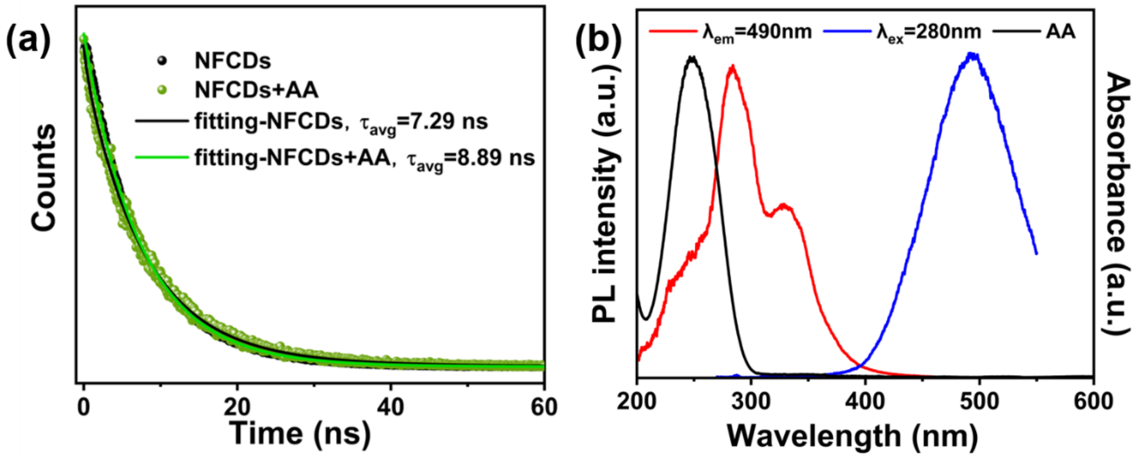

Figure S7 (a) The PL decay lifetime of NFCDs solution with and without the presence of AA. **(b)** UV-vis spectra of Fe^{3+} solution, the emission peaks excited at 280nm and the excitation peak at emission of 490 nm of NFCDs solution.

Reference:

1. Nagaraj, M.; Ramalingam, S.; Murugan, C.; Aldawood, S.; Jin, J.O.; Choi, I.; Kim, M., Detection of Fe^{3+} ions in aqueous environment using fluorescent carbon quantum dots synthesized from endosperm of Borassus flabellifer. *Environ. Res.* **2022**, *212*, 113273-113280.
2. Yu, J.; Xu, C.; Tian, Z.; Lin, Y.; Shi, Z., Facilely synthesized N-doped carbon quantum dots with high fluorescent yield for sensing Fe^{3+}. *New J. Chem.* **2016**, *40*, 2083-2088.
3. Cui, J.; Zhu, X.; Liu, Y.; Liang, L.; Peng, Y.; Wu, S.; Zhao, Y., N-Doped Carbon Dots as Fluorescent "Turn-Off" Nanosensors for Ascorbic Acid and Fe^{3+} Detection. *ACS Appl. Nano Mater.* **2022**, *5*, 7268-7277.
4. Liu, Z.; Jia, R.; Chen, F.; Yan, G.; Tian, W.; Zhang, J.; Zhang, J., Electrochemical process of early-stage corrosion detection based on N-doped carbon dots with superior Fe^{3+} responsiveness. *J. Colloid Interface Sci.* **2022**, *606*, 567-576.
5. Song, Y.; Zhu, C.; Song, J.; Li, H.; Du, D.; Lin, Y., Drug-Derived Bright and Color-Tunable N-Doped Carbon Dots for Cell Imaging and Sensitive Detection of Fe^{3+} in Living Cells. *ACS Appl. Mater. Interfaces* **2017**, *9*, 7399-7405.
6. Zhao, P.; Zhang, Q.; Cao, J.; Qian, C.; Ye, J.; Xu, S.; Zhang, Y.; Li, Y., Facile and Green Synthesis of Highly Fluorescent Carbon Quantum Dots from Water Hyacinth for the Detection of Ferric Iron and Cellular Imaging. *Nanomaterials* **2022**, 12, 1528-1536.
7. Ye, S.; Zhang, M.; Guo, J.; Song, J.; Zeng, P.; Qu, J.; Chen, Y.; Li, H., Facile Synthesis of Green Fluorescent Carbon Dots and Their Application to Fe^{3+} Detection in Aqueous Solutions. *Nanomaterials* **2022**, 12, 1487-1502.
8. Xu, X.; Ren, D.; Chai, Y.; Cheng, X.; Mei, J.; Bao, J.; Wei, F.; Xu, G.; Hu, Q.; Cen, Y, Dual-emission carbon dots-based fluorescent probe for ratiometric sensing of Fe(III) and pyrophosphate in biological samples. *Sens. Actuators B Chem.* **2019**, 298, 126829-126834.
9. Bai, L.; Yan, H.; Feng, Y.; Feng, W.; Yuan, L., Multi-excitation and single color emission carbon dots doped with silicon and nitrogen: Synthesis, emission mechanism, Fe3+ probe and cell imaging. Chem. Eng. J. 2019, 373, 963-972.
10. Hu, G.; Ge, L.; Li, Y.; Mukhtar, M.; Shen, B.; Yang, D.; Li, J., Carbon dots derived from flax straw for highly sensitive and selective detections of cobalt, chromium, and ascorbic acid. *J. Colloid Interface Sci.* **2020**, *579*, 96-108.
11. Li, C.; Zeng, J.; Guo, D.; Liu, L.; Xiong, L.; Luo, X.; Hu, Z.; Wu, F., Cobalt-Doped Carbon Quantum Dots with Peroxidase-Mimetic Activity for Ascorbic Acid Detection through Both Fluorometric and Colorimetric Methods. *ACS Appl. Mater. Interfaces* **2021**, *13*, 49453-49461.
12. Gan, L.; Su, Q.; Chen, Z.; Yang, X., Exploration of pH-responsive carbon dots for detecting nitrite and ascorbic acid. *Appl. Surf. Sci.* **2020**, *530*, 147269-147276.
13. Liu, Y.; Wu, P.; Wu, X.; Ma, C.; Luo, S.; Xu, M.; Li, W.; Liu, S., Nitrogen and copper (II) co-doped carbon dots for applications in ascorbic acid determination by non-oxidation reduction strategy and cellular imaging. Talanta **2020**, *210*, 120649-120657.
